# Measurement and modeling of *clemastine fumarate* (antihistamine drug) solubility in supercritical carbon dioxide

**DOI:** 10.1038/s41598-021-03596-y

**Published:** 2021-12-21

**Authors:** Gholamhossein Sodeifian, Chandrasekhar Garlapati, Fariba Razmimanesh, Marziehsadat Ghanaat-Ghamsari

**Affiliations:** 1 Department of Chemical Engineering, Faculty of Engineering, University of Kashan, 87317-53153 Kashan, Iran; 2 Laboratory of Supercritical Fluids and Nanotechnology, University of Kashan, 87317-53153 Kashan, Iran; 3 Modeling and Simulation Centre, Faculty of Engineering, University of Kashan, 87317-53153 Kashan, Iran; 4 Department of Chemical Engineering, Puducherry Technological University, Puducherry, 605014 India

**Keywords:** Chemical engineering, Chemical engineering

## Abstract

The solubilities of *clemastine fumarate* in supercritical carbon dioxide (ScCO_2_) were measured for the first time at temperature (308 to 338 K) and pressure (12 to 27 MPa). The measured solubilities were reported in terms of mole faction (mol/mol total) and it had a range from 1.61 × 10^–6^ to 9.41 × 10^–6^. Various models were used to correlate the data. The efficacy of the models was quantified with corrected Akaike’s information criterion (AICc). A new cluster salvation model was derived to correlate the solubility data. The new model was able to correlate the data and deviation was 10.3% in terms of average absolute relative deviation (AARD). Furthermore, the measured solubilities were also correlated with existing K.-W. Chen et al., model, equation of state model and a few other density models. Among density models, Reddy and Garlapati model was observed to be the best model and corresponding AARD was 7.57% (corresponding AIC_c_ was − 678.88). The temperature independent Peng–Robinson equation of state was able to correlate the data and AARD was 8.25% (corresponding AIC_c_ was − 674.88). Thermodynamic parameters like heats of reaction, sublimation and solvation of *clemastine fumarate* were calculated and reported.

## Introduction

The *clemastine fumarate* is a special drug and it has specific uses. It is an antihistamine with antimuscarinic and partial sedative properties. One of its forms also acts an antileishmanial drug. It also stimulates a macrophage response to leishmaniainfection^1^. For all the medical studies (for both in vivo and in vitro) a proper dosage is very essential and this may be achieved through proper particle size^1^. The usage of supercritical fluid technology in particle micronisation has gained significant importance in the recent times, wherein, carbon dioxide as a supercritical fluid has been used widely in practice^2^. The application of carbon dioxide as supercritical fluid solvent has several advantages over conventional solvents^2^ and it is designated as ScCO_2_. It possesses attractive physical properties such as, gas like diffusivity and liquid like density with low viscosity and surface tension^2,3^. By adjusting pressures and temperatures, one can tune the density of ScCO_2_ as desired and it is exploited in various applications. Due to this tunable nature, it has been used as a solvent in various process applications. ScCO_2_’s major applications include drug particle micronization, extraction, reactions, food processing, textile dyeing, ceramic coating, and many more^4–8^. To implement SFT, one needs to have exact phase equilibrium information such as saturation solubility. Solubility is one of the basic information that is essential for the design and development of SFT. Drug particle micronization requires precise solubility information and in literature, solubility of many solid drugs^9,10^ in ScCO_2_ is readily available, however, the solubility of *clemastine fumarate* is not reported. Therefore, for the first time, the solubility of *clemastine fumarate* in ScCO_2_ is reported in this work. We believe that this study may be useful in particle micronization using ScCO_2_.

The main objectives of the present work are in two stages; in the first stage we determine solubility of *clemastine fumarate.* Since, measuring experimental solubility data at each pressure and temperature is very difficult, we need a proper model to generate the solubility data^11^. Thereby, in the second stage we have developed a new cluster solubility model. The proposed model is compared with existing cluster solvation model. Furthermore, few density models and equation of state model are evaluated.

## Experimental

### Materials

**Figure 1:**
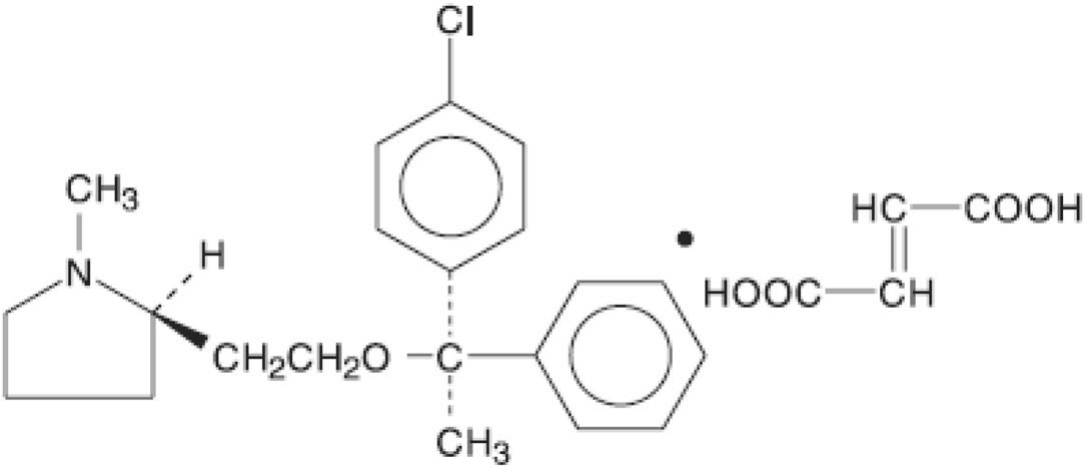
Chemical structure of *clemastine fumarate.*

Gaseous CO_2_ (purity > 99.9%) was obtained from Fadak company, Kashan (Iran), *clemastine fumarate* (CAS Number: 14976-57-9, purity > 99%) was obtained from Amin Pharma company. Methanol (CAS No. 67-56-1, purity > 99.9%) was obtained from Sigma Aldrich company. Table 1 indicates all the information about the chemicals utilized in this work. The molecular formula of *clemastine fumarate* is C_21_H_26_ClNO·C_4_H_4_O_4_ and its molecular weight is 459.97. The chemical structure is shown in Fig. 1.

**Table 1:** Basic properties of the used materials.

Compound	Formula	M_W_ (g/mol)	T_m_ (K)	λ_max_ (nm)	CAS number	Minimum purity by supplier (%)
Clemastine Fumarate	C_21_H_26_ClNO·C_4_H_4_O_4_	459.96	451.15	270	14976-57-9	99
Carbon dioxide	CO_2_	44.01			124-38-9	99.99
Methanol	CH_3_OH	32.04			67-56-1	99.9

### Experiment details

**Figure 2:**
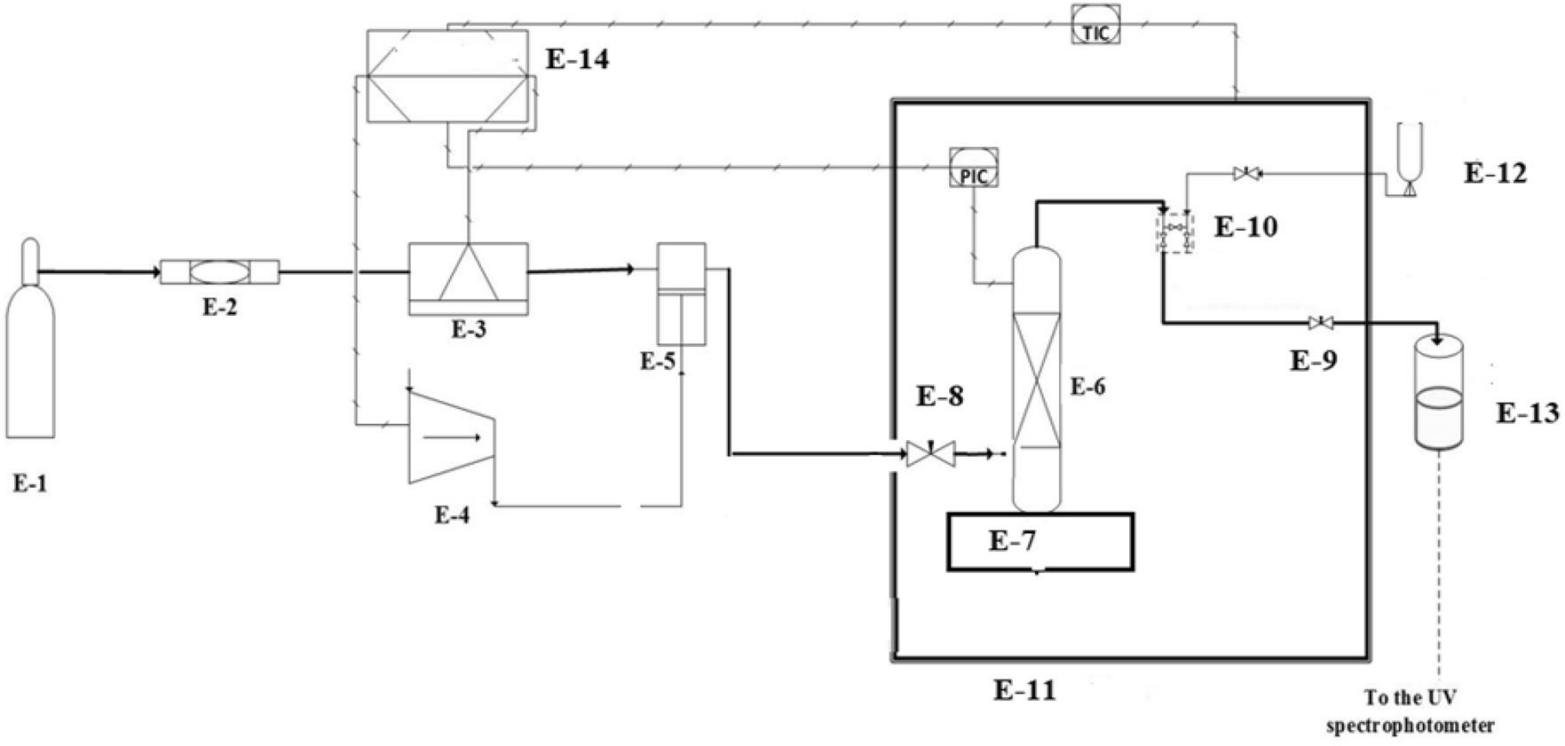
Line diagram of the solubility measurement device, E-1—CO_2_ cylinder; E-2—Filter; E-3—Refrigerator unit; E-4—Air compressor; E-5—High pressure pump; E-6—Equilibrium cell; E-7—Magnetic stirrer; E-8—Needle valve; E-9—Back-pressure valve; E-10—Six-port, two position valve; E-11—Oven; E-12—Syringe; E13—Collection vial; E-14—Control panel.

Figure 2 shows the line diagram of the equipment used in the study. More details about the solubility measuring device have been presented in our earlier studies^12–20^. However, a brief outline of the same has been presented in this section. This measuring methodology may be classified as an isobaric-isothermal method^21^. Each reading has been reported by controlling temperature and pressure at desired values within ± 0.1 K and ± 0.1 MPa precision, respectively. For each experiment about 1 g of *clemastine fumarate* drug has been used in the static cell. The saturation samples have been collected from the static cell after equilibrating for 60 min. Our earlier studies indicated that 60 min is enough for equilibrium. After equilibrium, saturated ScCO_2_ samples (600 µL) have been collected via 2-status 6-way port valve in a methanol preloaded vial. Once a sample was collected, the port valve was washed with 1 mL methanol. Thus, the total saturation solution obtained was 5 mL. Each measurement has been repeated thrice and average readings were reported. For calculations, the following formulas have been used^12–20^.

1 $$y_{2} = \frac{{n_{drug} }}{{n_{drug} + n_{{CO_{2} }} }}$$

where ${n}_{\text{drug}}$ denotes the quantity of the drug, and ${n}_{{\text{CO}}_{2}}$ denotes the quantity of CO_2_ in the sampling loop.

Further, we quantify moles of drug and moles of CO_2_ as

2 $${n}_{\text{drug}}=\frac{{C}_{s}\cdot {V}_{s}}{{M}_{s}}$$

3 $${n}_{{\text{CO}}_{2}}=\frac{{V}_{1}\cdot {\rho }_{1}\cdot }{{M}_{{\text{CO}}_{2}}}$$

where ${C}_{\text{s}}$ denotes the drug concentration in saturated sample vial in g/L. The volume of sampling loop, V_s_ = 5 × 10^–3^ m^3^ and vial collection, V_1_ = 600 × 10^–6^ m^3^. The $M_{s}$ and $M_{{CO_{2} }}$ denote the molecular weight of drug and CO_2_, respectively. Solubility is also described as

4 $$S = \frac{{C_{S} V_{s} }}{{V_{1} }}$$

The relation between S and $y_{2}$ is explained as

5 $$S = \frac{{\rho M_{s} }}{{M_{{CO_{2} }} }}\frac{{y_{2} }}{{1 - y_{2} }}$$

To ensure equilibrium solubility, the experiments were performed with fresh samples at various time intervals. For a specified temperature and pressure in each experiment, the drug sample was contacted with ScCO_2_ and stirred thoroughly in an equilibrium cell until a specific time (5 min, 10 min, 20 min, 30 min, 40 min, 50 min and 60 min) and the solubility readings were recorded. It was observed that the solubility was independent of time after 30 min. This experimental setup has already been validated in our previous works with alpha-tocopherol and naphthalene^17^.

A UV–visible (UNICO-4802) spectrophotometer has been used for the measurements of *clemastine fumarate* solubility. Samples collected for analysis in methanol solvent were analyzed at 270 nm.

## Models

In this section, a brief note about the existing density models and their mathematical form were presented.

### Existing empirical and semi-empirical models

#### Alwi–Garlapati model^22^

It is a semi-empirical model. It has three parameters. According to this, solubility is represented as a function of reduced temperature and reduced density and it is mathematically stated as

6 $$y_{2} = \frac{1}{{\rho_{1r} T_{r} }}\exp \left( {A_{1} + \frac{{A_{2} }}{{T_{r} }} + A_{3} \rho_{1r} } \right)$$

where $A_{1} - A_{3}$ are model constants.

#### Bartle et al., model^23^

It is based on enhancement factor concept and it has three parameters. According to this, solubility is represented as a function of pressure, temperature and density and it is mathematically stated as

7 $$\ln \left( {\frac{{y_{2} P}}{{P_{ref} }}} \right) = B_{1} + \frac{{B_{2} }}{T} + B_{3} \left( {\rho_{1} - \rho_{ref} } \right)$$

where $B_{1} - B_{3}$ are model constants. From parameter $B_{2}$ one can estimate sublimation enthalpy using the relation, $\Delta_{sub} H = - B_{2} R$ in which *R* is universal gas constant. Reference pressure (P_ref_) and density $\rho_{ref}$ are 0.1 MPa and 700 kg/m^3^, respectively.

#### Bian et al., model^24^

It is a five parameter model. It is an empirical model and it is mathematically stated as

8 $$y_{2} = \rho_{1}^{{\left( {D_{1} + D_{2} \rho_{1} } \right)}} \exp \left( D_{3}/T + D_{4}\rho_{1}/T + D_{5} \right)$$

where $D_{3} - D_{5}$ are model constants.

#### Chrastil model^25^

It is a three parameter model. It is a semi empirical model and it is mathematically stated as

9 $$c_{2} = \rho_{1}^{\kappa } \exp \left( {E_{1} + \frac{{E_{2} }}{T}} \right)$$

where $\kappa ,E_{1} \;{\text{and}}\;E_{2}$ are model constants.

In terms of mole fraction it is mathematically stated as

9a $$y_{2} = \frac{{\left( {\rho_{1} } \right)^{\kappa - 1} \exp \left( {E_{1} + \frac{{E_{2} }}{T}} \right)}}{{\left[ {1 + \left( {\rho_{1} } \right)^{\kappa - 1} \exp \left( {E_{1} + \frac{{E_{2} }}{T}} \right)} \right]}}$$

#### Reformulated Chrastil model^26,27^

It is also a three parameter model. It is a semi-empirical model and it is mathematically stated as

10 $$y_{2} = \left( {\frac{{RT\rho_{1} }}{{M_{CF} f^{ \cdot } }}} \right)^{{\kappa^{\prime } - 1}} \exp \left( {F_{1} + \frac{{F_{2} }}{T}} \right)$$

where $\kappa^{\prime } ,F_{1} \;{\text{and}}\;F_{2}$ are model constants. Reference fugacity ($f^{ * }$) is 0.1 MPa.

#### Garlapati–Madras model^28^

It is a five parameter model. It is a mathematical model and it is mathematically stated as

11 $$\ln \left( {y_{2} } \right) = G_{1} + (G_{2} + G_{3} \rho_{1} )\ln \left( {\rho_{1} } \right) + \frac{{G_{4} }}{T} + G_{5} \ln \left( {\rho_{1} T} \right)$$

where $G_{1} - G_{5}$ are model constants.

#### Mendez–Teja model (MT model)^29^

It is a semi-empirical model and it has three parameters. It is mathematically stated as

12 $$T\ln \left( {y_{2} P} \right) = H_{1} + H_{2} \rho_{1} + H_{3} T$$

where $H_{1} - H_{3}$ are model constants.

Equation (12) is used in checking self-consistency of the measured solubility data. Accordingly, all the data points lie on a line when they are plotted $T\ln \left( {y_{2} P} \right) - H_{3} T$ versus $\rho_{1}$.

#### Sodefian et al., model^30^

It is a mathematical model and it has six parameters and it is mathematically stated as

13 $$\ln \left( {y_{2} } \right) = I_{1} + \frac{{I_{2} P^{2} }}{T} + I_{3} \ln \left( {\rho_{1} T} \right) + I_{4} \left( {\rho_{1} \ln \left( {\rho_{1} } \right)} \right) + I_{5} P\ln \left( T \right) + I_{6} \frac{{\ln \left( {\rho_{1} } \right)}}{T}$$

where $I_{1} - I_{9}$ are model constants.

#### Reddy–Garlapati model^9^

It is based on degree of freedom. It is a six parameter model. It is an empirical model and it is mathematically stated as

14 $$y_{2} = \left( {J_{1} + J_{2} P_{r} + J_{3} P_{r}^{2} } \right)T_{r}^{2} + (J_{4} + J_{5} P_{r} + J_{6} P_{r}^{2} )$$

where $J_{1} - J_{6}$ are model constants.

#### Mahesh–Garlapati model^11^

It is based on degree of freedom. It is a three parameter model. It is an empirical model and it is mathematically stated as

15 $$y_{2} = \exp \left( {K_{1} + K_{2} \rho_{1r} T_{r} + K_{3} \rho_{1r} T_{r}^{3} } \right)$$

### Equation of state (EoS) model

The solubility of *clemastine fumarate* drug, $i$ (solute), in a supercritical carbon dioxide, $j$(solvent), is expressed as^31^

16 $${\text{y}}_{{\text{i}}} = \frac{{p_{i}^{S} \hat{\phi }_{i}^{S} }}{{P\hat{\phi }_{i}^{{ScCO_{2} }} }}\exp \left[ {\frac{{\left( {P - p_{i}^{S} } \right)v_{i} }}{RT}} \right] \,$$

where $p_{i}^{s}$ is solute sublimation pressure;$v_{i}$ is solute molar volume; The fugacity coefficient of the pure solute at saturation ($\hat{\phi }_{i}^{S}$) is usually taken to be unity. In this work, $\hat{\phi }_{i}^{{ScCO_{2} }}$ is the fugacity coefficient of the solute in the solvent phase. $\hat{\phi }_{i}^{{ScCO_{2} }}$ is calculated using Peng–Robinson (PR) EoS along with two parameter van der Waals mixing rule (vdW2)^32^. The expression used for calculation of $\hat{\phi }^{{ScCO_{2} }}$ is obtained from the following basic thermodynamic relation^33^.

17 $$\ln \left( {\hat{\varphi }_{i}^{{ScCO_{2} }} } \right) = \frac{1}{RT}\int\limits_{v}^{\infty } {\left[ {\left( {\frac{\partial P}{{\partial N_{i} }}} \right)_{{T,V,N_{j} }} - \frac{RT}{v}} \right]} dv - \ln Z$$

The general PREoS form^32^ is

18 $$P = \frac{RT}{{v - b}} - \frac{a\left( T \right)}{{v\left( {v + b} \right) + b\;\left( {v - b} \right)}} \,$$

The pure component parameters *a* and *b* are

19 $$a{\kern 1pt} \left( T \right) = 0.45724\frac{{R^{2} T_{c}^{2} }}{{P_{c} }}\left[ {1 + \left( {0.37464 + 1.5422\omega - 0.26992\omega^{2} \left( {1 - \sqrt {T_{r} } } \right)} \right)} \right] \,$$

20 $$b = 0.07780\frac{{RT_{c} }}{{P_{c} }} \,$$

The expression for $\hat{\phi }_{i}^{{ScCO_{2} }}$ is:

21 $$\begin{aligned} {\text{ln}}\left( {\hat{\phi }_{{\text{i}}}^{ScF} } \right) & = \frac{{\hat{b}}}{b} \, \left( {{\text{Z}} - {1}} \right) - {\text{ ln}}\left[ {{\text{Z}}\left( {{1 - }\frac{b}{v}} \right)} \right] \, + \frac{{\text{a}}}{{\left( {{2}\sqrt {2} } \right)bRT}}\left[ {\frac{{{\hat{\text{a}}}}}{a} - \frac{{\hat{b}}}{b}} \right]{\text{ ln}}\left( {\frac{Z - 0.414b}{{Z + 2.414b}}} \right) \\ \hat{a} & = \frac{1}{n}\frac{{\partial n^{2} a}}{{\partial n_{i} }} = 2\sum {x_{i} a_{ij} } ;\quad \hat{b} = \frac{\partial nb}{{\partial n_{i} }} = 2\sum {x_{i} b_{ij} - b} \\ \end{aligned}$$

The expressions for VdW2

22 $$a = \sum\limits_{i} {\sum\limits_{j} {x_{i} x_{j} } } a_{ij}$$

23 $$b = \sum\limits_{i} {\sum\limits_{j} {x_{i} x_{j} b_{ij} } }$$

24 $$a_{ij} = \left( {1 - k_{ij} } \right)\sqrt {a_{ii} a_{jj} }$$

25 $$b_{ij} = \left( {1 - l_{ij} } \right)\frac{{\left( {b_{ii} + b_{jj} } \right)}}{2}$$

The PR EoS regression may be carried out either temperature independent or temperature dependent. For temperature independent regression suitable sublimation expression is used. The general form^34,35^ used for the regression purpose is

26 $$R\ln \left( {p_{sub} } \right) = \beta + \frac{\gamma }{T} + \Delta_{sub} \delta \ln \left( {\frac{T}{298.15}} \right)$$

The regression directly results in binary interaction parameters along with sublimation pressure expression coefficients ($\beta /R$, $\gamma/R$ and $\Delta_{sub} \delta /R$) and from parameters $\gamma$ and $\Delta_{sub} \delta$ we can estimate sublimation pressure. The expression for sublimation enthalpy is

27 $$\Delta_{sub} H = - \gamma + \Delta_{sub} \delta \;T$$

### K.-W. Chen et al., cluster model^36^

According to the model, the formation of solvate complex $AB_{\kappa }$ is due to the reaction mentioned in Eq. (28), where A is solute and B is supercritical fluid.

28 $$A + \kappa^{\prime \prime } B \Leftrightarrow AB_{\kappa }$$

It is an equilibrium reaction and at equilibrium the following condition is satisfied.

29 $$\sum {\nu_{i} \overline{F}{}_{i}(T,P,z_{i} ) = 0}$$

where $\sum {}$ is summation; $\nu$ and $\overline{F}$ are stoichiometric coefficient and partial molar Gibbs energy, respectively.

In general the partial molar Gibbs energy for species is written as

30 $$\overline{F}_{i} \left( {T,P,z_{i} } \right) = \overline{F}_{i}^{o} \left( {T,P^{o} ,z_{i}^{o} } \right) + \left( {\overline{F}_{i} \left( {T,P,z_{i} } \right) - \overline{F}_{i}^{o} \left( {T,P^{o} ,z_{i}^{o} } \right)} \right)$$

where $P^{o}$ and $z_{i}^{o}$ are reference state pressure and composition of species “i”. The reference pressure is taken as critical pressure of the supercritical fluid and finally the expression for the equilibrium in terms of fugacity coefficients is

31 $$\ln \left( {\frac{{z_{{AB_{\kappa } }} }}{{z_{B}^{{\kappa^{\prime \prime } }} }}} \right) = k^{\prime \prime } \ln \left( {\frac{{\hat{\phi }_{B} \left( {T,P,z_{B} } \right)P}}{{\phi_{B} \left( {T,P_{c,Scf} } \right)P_{c,scf} }}} \right) + \frac{{V_{s} \left( {P - P_{c,scf} } \right)}}{RT} - \ln \left( {\frac{{\hat{\phi }_{{AB_{\kappa } }} \left( {T,P,z_{{AB_{\kappa } }} } \right)P}}{{\phi_{{AB_{\kappa } }} \left( {T,P_{c,scf} } \right)P_{c,scf} }}} \right) - \frac{{\Delta F^{rxn} \left( {T,P_{c,scf} } \right)}}{RT}$$

where $\Delta F^{rxn} \left( {T,P_{c,scf} } \right)$ is the change in Gibbs energy as a result of formation of solvate complex.

The model has two parameters $\kappa^{\prime \prime }$ and $\Delta F^{rxn} \left( {T,P_{c,scf} } \right)$. K.-W. Chen et al.^35^, used the following temperature dependent general form^36^ in place of $\Delta F^{rxn} \left( {T,P_{c,scf} } \right)$

32 $$\Delta F^{rxn} \left( {T,P_{c,scf} } \right) = a^{\prime } - b^{\prime }T$$

Thus the final model has $\kappa^{\prime \prime }$, $a^{\prime }$ and $b^{\prime }$ (three adjustable parameters).

The Eq. (31) is further simplified with the help of Taylor series on left hand side

33 $$\ln \left( {\frac{{z_{{AB_{\kappa } }} }}{{1 - \kappa^{\prime \prime } z_{{AB_{\kappa } }} }}} \right) = k\ln \left( {\frac{{\hat{\phi }_{B} \left( {T,P,z_{B} } \right)P}}{{\phi_{B} \left( {T,P_{c,Scf} } \right)P_{c,scf} }}} \right) + \frac{{V_{s} \left( {P - P_{c,scf} } \right)}}{RT} - \ln \left( {\frac{{\hat{\phi }_{{AB_{\kappa } }} \left( {T,P,z_{{AB_{\kappa } }} } \right)P}}{{\phi_{{AB_{\kappa } }} \left( {T,P_{c,scf} } \right)P_{c,scf} }}} \right) - \frac{{\Delta F^{rxn} \left( {T,P_{c,scf} } \right)}}{RT}$$

The experimental solubility and cluster mole fractions are related as^37,38^ follows

34 $$y = z_{AB_{\kappa }}/\left( 1 + \kappa^{\prime\prime}\;z_{AB_{\kappa }} \right)$$

The fugacity coefficient of the components and mixtures are evaluated with PR EoS. For fugacity coefficient calculations we need mixture properties and they are calculated with the help of solute, solvent and cluster volume and energy parameter. More details about these can be seen elsewhere^36–38^.

The cluster obeys the following mixing rules for volume and energy parameters

35 $$b_{{AB_{\kappa } }} = k^{\prime \prime } b_{B} + b_{A}$$

36 $$a_{{AB_{\kappa } }} = \left[ k^{\prime \prime } \left( {a_{B} b_{B} } \right)^{0.5} + \left( {a_{A} b_{A} } \right)^{0.5} \right]^{2}\left /b_{AB_{\kappa \kappa }} \right.$$

More details about PR EoS, fugacity coefficient of pure component and mixture can be seen in section “Equation of state (EoS) model” and literature^37,38^.

The final expression for the solubility is

37 $$y = \frac{{\exp \left[ {\kappa^{\prime \prime \prime } \ln \left( {\frac{{\hat{\phi }_{B} \left( {T,P,z_{B} } \right)P}}{{\phi_{B} \left( {T,P_{c,Scf} } \right)P_{c,scf} }}} \right) + \frac{{V_{s} \left( {P - P_{c,scf} } \right)}}{RT} - \ln \left( {\frac{{\hat{\phi }_{{AB_{\kappa } }} \left( {T,P,z_{{AB_{\kappa } }} } \right)P}}{{\phi_{{AB_{\kappa } }} \left( {T,P_{c,scf} } \right)P_{c,scf} }}} \right) - \frac{{\left( {a^{\prime \prime } - b^{\prime \prime } T} \right)}}{RT}} \right]}}{{1 + 2\kappa^{\prime \prime \prime } \exp \left[ {\kappa^{\prime \prime \prime } \ln \left( {\frac{{\hat{\phi }_{B} \left( {T,P,z_{B} } \right)P}}{{\phi_{B} \left( {T,P_{c,Scf} } \right)P_{c,scf} }}} \right) + \frac{{V_{s} \left( {P - P_{c,scf} } \right)}}{RT} - \ln \left( {\frac{{\hat{\phi }_{{AB_{\kappa } }} \left( {T,P,z_{{AB_{\kappa } }} } \right)P}}{{\phi_{{AB_{\kappa } }} \left( {T,P_{c,scf} } \right)P_{c,scf} }}} \right) - \frac{{\left( {a^{\prime } - b^{\prime } T} \right)}}{RT}} \right]}}$$

### New cluster model

This model is an extension to existing K.-W. Chen et al., model^36^. According to the model the formation of solvate complex $AB_{\kappa }$ is according to the reaction mention in Eq. (38), where A is solute and B is supercritical fluid.

38 $$A + \kappa^{\prime \prime \prime } B \Leftrightarrow AB_{\kappa }$$

For the solubility model development we have used all arguments similar to that of K.-W. Chen et al., model. The main difference between K–W. Chen et al. model and the new cluster model lies in selection of temperature dependent general form. The considered temperature dependent general form is ^39^

39 $$\Delta F^{rxn} \left( {T,P_{c,scf} } \right) = a^{\prime \prime } + b^{\prime \prime } T\ln (T) + c^{\prime \prime } T$$

Thus, the final model has four adjustable parameters $\kappa^{\prime \prime \prime }$,$a^{\prime \prime }$, $b^{\prime \prime }$ and $c^{\prime \prime }$.

Follow in K.-W. Chen et al., footsteps we get the final expression for the solubility as

40 $$y = \frac{{\exp \left[ {\kappa^{\prime \prime \prime } \ln \left( {\frac{{\hat{\phi }_{B} \left( {T,P,z_{B} } \right)P}}{{\phi_{B} \left( {T,P_{c,Scf} } \right)P_{c,scf} }}} \right) + \frac{{V_{s} \left( {P - P_{c,scf} } \right)}}{RT} - \ln \left( {\frac{{\hat{\phi }_{{AB_{\kappa } }} \left( {T,P,z_{{AB_{\kappa } }} } \right)P}}{{\phi_{{AB_{\kappa } }} \left( {T,P_{c,scf} } \right)P_{c,scf} }}} \right) - \frac{{\left( {a^{\prime \prime } + b^{\prime \prime } T\ln (T) + c^{\prime \prime } T} \right)}}{RT}} \right]}}{{1 + 2\kappa^{\prime \prime \prime } \exp \left[ {\kappa^{\prime \prime \prime } \ln \left( {\frac{{\hat{\phi }_{B} \left( {T,P,z_{B} } \right)P}}{{\phi_{B} \left( {T,P_{c,Scf} } \right)P_{c,scf} }}} \right) + \frac{{V_{s} \left( {P - P_{c,scf} } \right)}}{RT} - \ln \left( {\frac{{\hat{\phi }_{{AB_{\kappa } }} \left( {T,P,z_{{AB_{\kappa } }} } \right)P}}{{\phi_{{AB_{\kappa } }} \left( {T,P_{c,scf} } \right)P_{c,scf} }}} \right) - \frac{{\left( {a^{\prime \prime } + b^{\prime \prime } T\ln (T) + c^{\prime \prime } T} \right)}}{RT}} \right]}}$$

Hereafter it may be called as cluster model by Sodeifian et al. The major advantage of Eq. (40) over Eq. (37) lies in improved parameterization and efficacy.

For implementing EoS and cluster models we need critical properties and vapour pressures, and they are estimated with the help of group contribution methods. Critical temperature is estimated by Fedors method^40,41^, critical pressure is estimated by Joback modification of Lydersen’s method^41^. The acentric factor is estimated by Lee–Kesler vapour pressure relations. While calculating vapour pressure, the normal boiling temperature (at 1.0 atm) is required and it is estimated from Klincewicz relation, T_c_ = 50.2–0.16 M + 1.41 T_b_ were M is molecular weight^41^. The required molar volume of drug (solid) is estimated by Immirzi, A.; Perini, B method^42,43^ and the vapour pressures are estimated by Lee–Kesler vapour method^41^.

All the models mentioned in sections “Existing empirical and semi-empirical models”, “Equation of state (EoS) model”, “K.-W. Chen et al., cluster model^36^” and “New cluster model” are evaluated with the following objective function^44^.

41 $$OF = \sum\limits_{i = 1}^{N} {\left| {y_{i}^{\exp } - y_{i}^{cal}} \right|} \left/y_{i}^{\exp} \right.$$

Regression results are represented in terms of average absolute relative deviation percentage (AARD %)

42 $$AARD\% = 100/N \sum\limits_{i = 1}^{N} \left| {y_{i}^{\exp } - y_{i}^{cal}} \right|/{y_{i}^{\exp}}$$

where *N* is number of experimental data points; $y_{i}$ is mole fraction; the superscripts *cal* and *exp* denote thecalculated and measured mole fractions, respectively.

The correlating ability of a model depends on the number of its parameter. The Akaike’s information criterion (AIC)^45–49^ is used to assess the correlating efficacy of a model regardless of the number of its parameters.

43 $$AIC = N\ln (SSE/N) + 2N_{p}$$

where N is number of experimental data points; $N_{p}$ is model parameters; SSE is error sum of squares.

When N is less than 40 corrected AIC is used and it is stated as follows

44 $$AIC_{c} = AIC + 2N_{p} \left( N_{p} + 1 \right)/\left( N - N_{p} - 1 \right)$$

## Results and discussion

**Figure 3:**
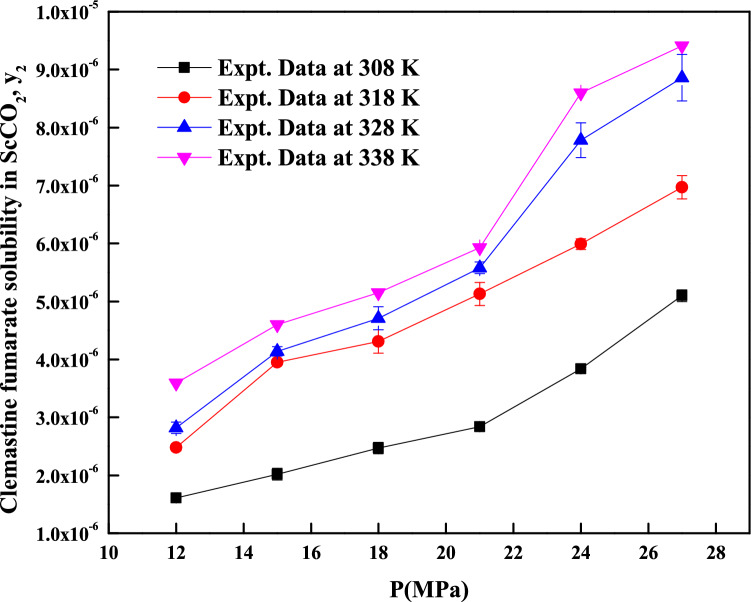
*Clemastine fumarate*solubility in ScCO_2_ and effect of pressure on isotherms.

**Figure 4:**
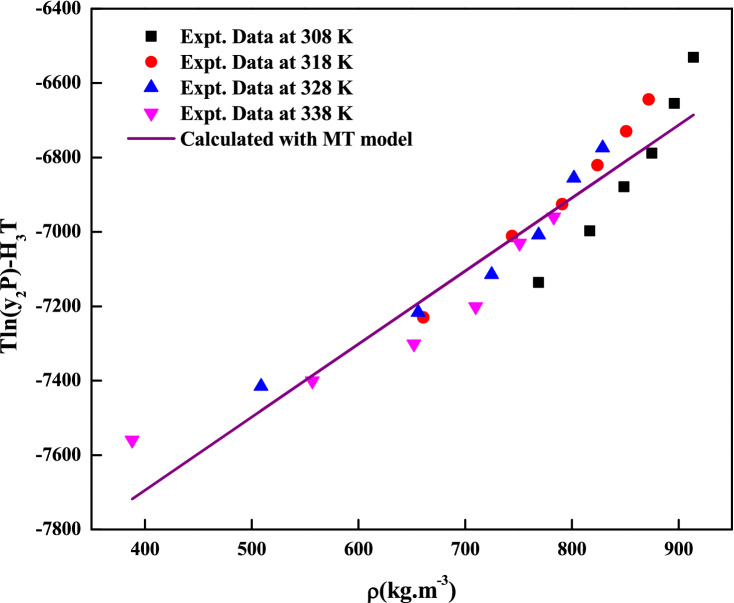
Self-consistency plot of *clemastine fumarate* solubility in ScCO_2_. Symbols are experimental points; line is calculated with MT Model.

Table 1 indicates some properties of the used materials. Table 2 shows *clemastine fumarate* solubility in ScCO_2_. The density indicated in Table 2 is obtained from the NIST data base^50^. Computed properties of *clemastine fumarate* are shown in Table 3. Figure 3 indicates the effect of pressure on various isotherms and no cross over region observed, such solubility behavior is observed for some other pharmaceutical compounds in our earlier studies^14^. From Table 3, it is clear that the vapor pressure of *clemastine fumarate* increases from 0.0114 Pa to 0.1277 Pa, when the temperature is increased from 308 to 338 K, it is a 11.2 fold jump. Due to this, solubility increases from 0.0161 × 10^–4^ to 0.0359 × 10^–4^ (in mole/mole total) at 12 MPa (it is a 2.23 fold jump) and 0.051 × 10^–4^ to 0.0941 × 10^–4^ (in mole/mole total) at 27 MPa (it is a 1.845 fold jump). At the same time, densities have changed from 769 kg m^-3^ (corresponding to 308 K and 12 MPa) to 338 kg m^-3^ (corresponding to 338 K and 12 MPa) and 914 kg m^-3^ (corresponding to 308 K and 27 MPa) to 783 kg m^-3^ (corresponding to 338 K and 27 MPa),which clearly indicates that density decreases at 12 MPa (i.e., 338/769 = 0.4395) and somewhat increases at 27 MPa (i.e., 783/914 = 0.8567). From preceding arguments we say that the pressure effect is less pronounced with respect to density than the temperature effect. This kind of nonlinearity is well captured with models having more parameters compared to less number of parameter^14^. Therefore, models proposed by Sodeifian et al. model and Reddy–Garlapati model are able to correlate the solubility in a better manner. Figure 4 indicates the self-consistency of the measured data with MT model.

**Table 2:** Solubility of *Clemastine Fumarate* in ScCO_2_ at various temperatures and pressures (the experimental standard deviation was obtained by $S(y_{k} ) = \sqrt {\frac{{\sum\nolimits_{j = 1}^{n} {(y_{j} - \overline{y})^{2} } }}{n - 1}}$. Expanded uncertainty (U) = k*u_*combined*_ and the relative combined standard uncertainty *u*_*combined*_*/y* = $\sqrt {\sum\nolimits_{i = 1}^{N} {(P_{i} u(x_{i} )/x_{i} )^{2} } }$.

Temperature (K)^a^	Pressure (MPa)^a^	Density of SC-CO_2_ (kg/m^3^) [2]	y_2_ × 10^4^ (Mole fraction)	Experimental standard deviation, S(ȳ) × (10^4^)	S (equilibrium solubility) (g/L)	Expanded uncertainty of Mole fraction (10^4^ U)
308	12	769	0.0161	0.0005	0.0130	0.0012
	15	817	0.0202	0.0010	0.0173	0.0022
	18	849	0.0247	0.0010	0.0219	0.0023
	21	875	0.0284	0.0008	0.0260	0.0020
	24	896	0.0384	0.0002	0.0360	0.0017
	27	914	0.051	0.0010	0.0488	0.0030
318	12	661	0.0248	0.0006	0.0171	0.0017
	15	744	0.0395	0.0005	0.0307	0.0021
	18	791	0.0431	0.0020	0.0357	0.0044
	21	824	0.0513	0.0020	0.0442	0.0046
	24	851	0.0599	0.0009	0.0532	0.0032
	27	872	0.0697	0.0020	0.0636	0.0050
328	12	509	0.0282	0.0010	0.0150	0.0024
	15	656	0.0414	0.0008	0.0284	0.0025
	18	725	0.0471	0.0020	0.0357	0.0045
	21	769	0.0558	0.0010	0.0449	0.0032
	24	802	0.0778	0.0030	0.0652	0.0069
	27	829	0.0886	0.0040	0.0767	0.0089
338	12	388	0.0359	0.0010	0.0145	0.0026
	15	557	0.046	0.0020	0.0268	0.0045
	18	652	0.0515	0.0007	0.0351	0.0027
	21	710	0.0593	0.0010	0.0440	0.0033
	24	751	0.086	0.0040	0.0676	0.0087
	27	783	0.0941	0.0030	0.0771	0.0073

^a^Standard uncertainty u are u(T) =  ± 0.1 K; u(p) =  ± 0.1 MPa. The value of the coverage factor k = 2 was chosen on the basis of the level of confidence of approximately 95 percent.

**Table 3:** Properties of *Clemastine fumarate* and CO_2_^a^.

Substance	Tc (K)	Pc (MPa)	$\omega$	V^s^ × 10^–4^ (m^3^/mol)	T (K)			
					P_sub_ (Pa)^f^			
					308	318	328	338
Clemastine Fumarate	901.25^b^	1.409^c^	0.337^d^	364.764^e^	0.0114	0.02699	0.0603	0.1277
CO_2_	304.18	7.38	0.225					

^a^Critical temperature: T_c_; Critical pressure: P_c_; Acentric factor: *ω*; Solid molar volume: Vs; Temperature: T.

^b^Estimated by Fedors method^40,41^.

^c^Estimated by the Joback modification of Lydersen’s method^41^.

^d^Estimated by Lee–Kesler vapour pressure relations. (Note: The required normal boiling temperature (at 1.0 atm), T_b_ is estimated with Klincewicz relation, T_c_ = 50.2–0.16 M + 1.41 T_b_ were M is molecular weight)^41^.

^e^Estimated by Immirzi, A.; Perini, B method^42,43^.

^f^Estimated by Lee–Kesler vapour method^41^.

**Figure 5:**
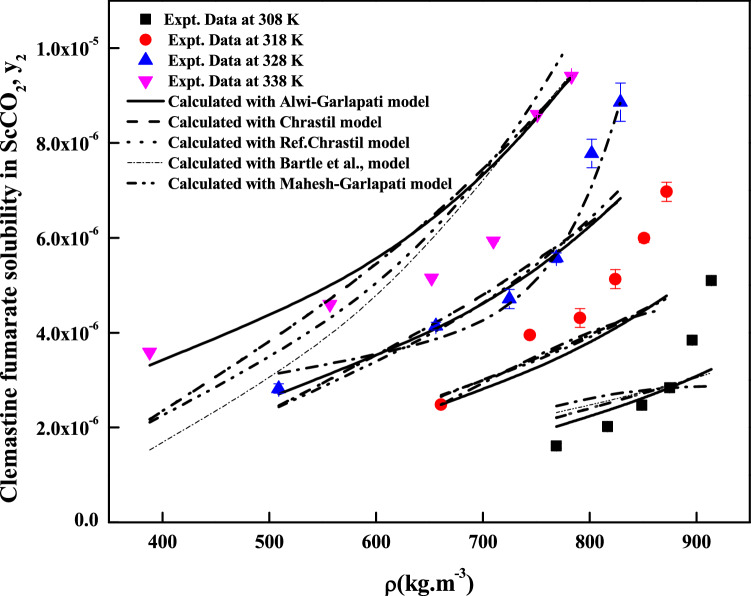
Solubility of*clemastine fumarate* in ScCO_2_. Symbols are experimental points; lines are calculated with three parameter models.

**Figure 6:**
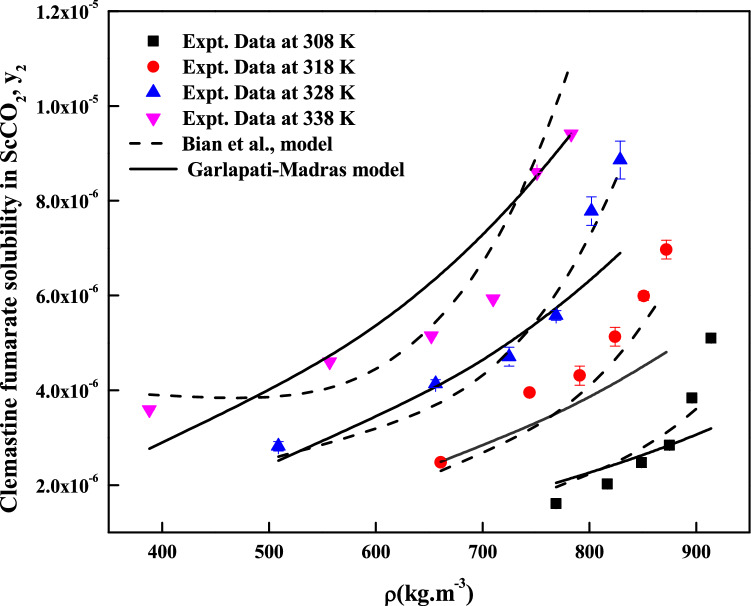
Solubility of *clemastine fumarate* in ScCO_2_. Symbols are experimental points; lines are calculated with five parameter models.

**Figure 7:**
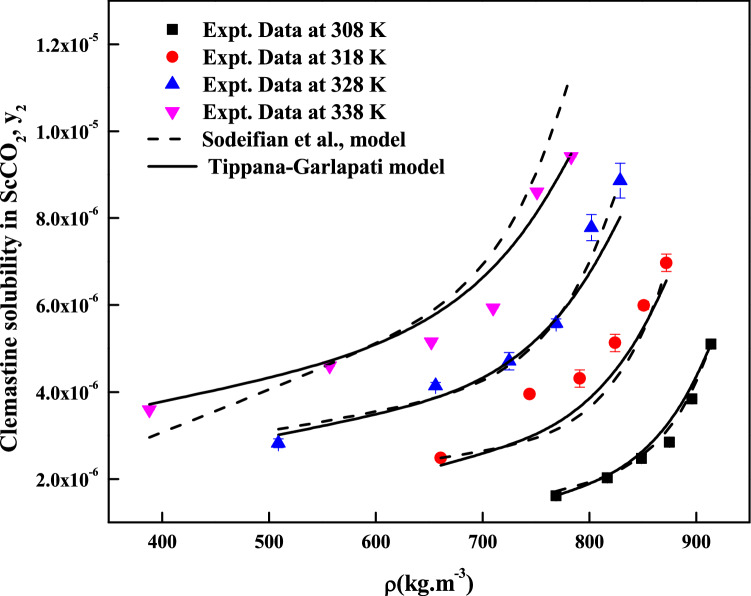
Solubility of *clemastine fumarate* in ScCO_2_. Symbols are experimental points; lines are calculated with six parameter models.

**Figure 8:**
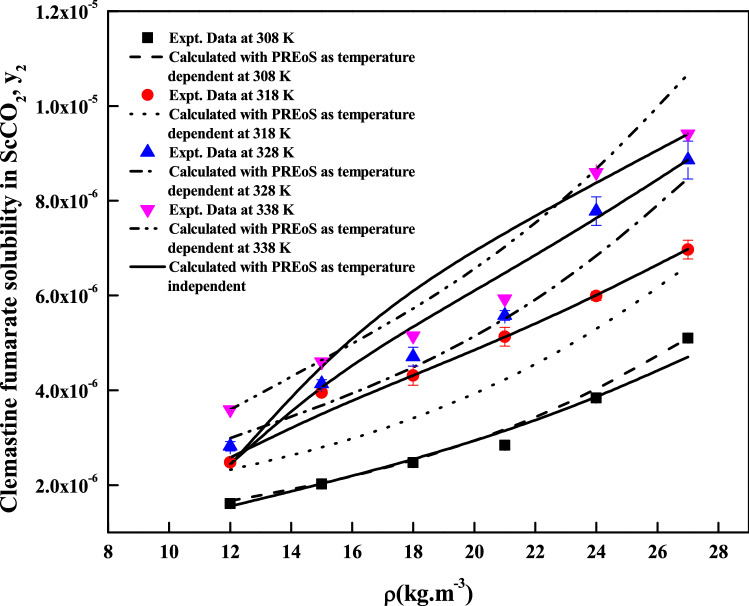
Solubility of *clemastine fumarate* in ScCO_2_. Symbols are experimental points; lines are calculated with PREoS models.

**Figure 9:**
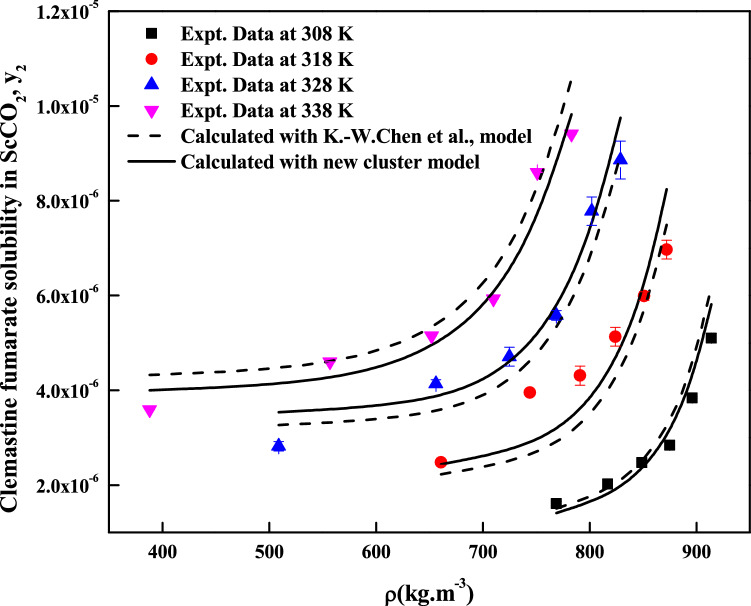
Solubility of *clemastine fumarate* in ScCO_2_. Symbols are experimental points; lines are calculated with cluster models.

The regression analysis of experimental data is carried out easily with density model, but the regression analysis of EoS model and cluster model requires critical properties of the solute and solvent. The required critical temperature, critical pressure, acentric factor and molar volume of the solute and sublimation pressure of the solute are not readily available; due to this these properties are computed with standard group contribution methods^39–42^. The empirical and semi empirical models considered in this study have shown different degree of fitting in terms of AARD%. The regression results of various models are indicated in Tables 4, 5 and 6. Among the existing empirical and semi-empirical models, Reddy–Garlapati model is having lower AARD%. Chrastil model parameter ($E_{2}$) and Reformulated Chrastil model parameter ($F_{2}$) are used in calculating total enthalpy, from Bartel et al., model parameter ($B_{2}$) we get sublimation enthalpy of the *clemastine fumarate*. Heat of solvation is obtained from the magnitude difference between total enthalpy and sublimation enthalpy. The more details about these calculations can be seen in literature^34^. All the computed results are reported in Table 7. EoS model is regressed in two different ways. In the first approach correlation parameter are treated as temperature dependent where as in second approach the correlation parameters are treated as temperature independent. From regression results (Table 5) temperature independent correlation is better than temperature dependent correlation. EoS model also provide sublimation enthalpy and it is reported in Table 7. From Table 6 it is clear that the cluster model by sodeifian et al., is superior to exiting K.-W. Chen et al., model. The parameters ‘$a^{\prime \prime }$’ and ‘$b^{\prime \prime }$’ of cluster model by sodeifian et al., directly results in enthalpy change and entropy change of the cluster formation process. The positive sign for entropy change indicates an increase in disorder. The positive change in enthalpy indicates heat absorption from surroundings the by the reaction. The correlating ability of the various models is represented in Figs. 4, 5, 6, 7, 8 and 9.

**Table 4:** Correlation constants for the exiting empirical models.

Model		Correlation parameters		AARD%	R^2^
Alwi–Garlapati model		*A*_1_ = 1.8759; *A*_2_ = − 18.105; *A*_3_ = 2.0667		14.00	0.809
Bartel et al., model		*B*_1_ = 14.719; *B*_2_ = − 7179.9; *B*_3_ = 6.6739 × 10^–3^		20.2	0.765
Bian et al., model		$D_{1}$ = − 4.9614; $D_{2}$ = 5.5723 × 10^–3^; $D_{3}$ = 2030.5; $D_{4}$ = − 9.9873; $D_{5}$ = 9.6915		11.2	0.927
Chrastil model		$\kappa$ = 3.0938; $E_{1}$ = − 11.003; $E_{2}$ = − 4907.2		16.7	0.785
Ref. Chrastil model		$\kappa^{\prime}$ = 3.0813; $F_{1}$ = − 21.619; $F_{2}$ = − 4216.6		16.7	0.784
Garlapati–Madras model		$G_{1}$ = − 755.12; $G_{2}$ = 859.01; $G_{3}$ = 0.9875; $G_{4}$ = − 8597.4; $G_{5}$ = − 10.722		14.6	0.818
Mendez–Teja model		$H_{1}$ = − 8479.4; $H_{2}$ = 1.9629; $H_{3}$ = 14.617		21.69	0.706
Sodeifian et al., model		$I_{1}$ = − 42.487; $I_{2}$ = − 6.9315 × 10^–4^; $I_{3}$ = 2.4265; $I_{4}$ = − 4.2127 × 10^–4^; $I_{5}$ = 1.929 × 10^–2^; $I_{6}$ = 62.052		8.78	0.929
Tippana–Garlapati model		$J_{1}$ = 8.334 × 10^–7^; $J_{2}$ = 1.3157 × 10^–5^; $J_{3}$ = − 3.3583 × 10^–7^; $J_{4}$ = 5.6805 × 10^–7^; $J_{5}$ = − 1.3913 × 10^–5^; $J_{6}$ = 7.7736 × 10^–7^		7.57	0.951
Mahesh–Garlapati model		$K_{1}$ = − 14.614; $K_{2}$ = − 2.4145; $K_{3}$ = 3.3127		17.9	0.797

**Table 5:** Correlation constants of PR EoS + VdW2 combination.

Model	Correlation parameters	T = 308 K	T = 318 K	T = 328 K	T = 338 K
**Temperature dependent parameters**					
PREoS-VdW2 temperature dependent parameters	$k_{ji}$	0.58814	0.55098	0.55315	0.53741
	$l_{ji}$	0.5856	0.52813	0.52218	0.48034
	AARD%	3.99	2.5572	7.5542	13.067
**Temperature independent parameters**					
PREoS-VdW2 temperature independent parameters	$k_{ji}$	0.79788			
	$l_{ji}$	0.74029			
	$\beta /R$	0.27409			
	$\gamma /R$	− 221.54			
	$\Delta_{sub} \delta /R$	11.305			
	AARD%	8.2458

**Table 6:** Correlation constants of cluster models.

Model	Correlation parameters	AARD%	R^2^
New model	$\kappa^{\prime \prime\prime }$ = 0.10756; $a^{\prime\prime }$ = 443,590; $b^{\prime\prime }$ = 1357.1; $c^{\prime\prime }$ = − 9115.7	10.3	0.936
K.-W. Chen et al., model	$\kappa^{\prime \prime }$ = 0.10794; $a^{\prime }$ = 6093.7; $b^{\prime }$ = − 70.319	12.1	0.913

**Table 7:** Summary of thermodynamic properties.

Model	Property		
	Total enthalpy, ΔH_total_ (kJ/mol)	Enthalpy of sublimation ΔH_sub_ (kJ/mol)	Enthalpy of solvation,$\Delta H_{sol}$ (kJ/mol)
Chrastil model	40.798^a^		− 18.896^e^; − 12.282^f^
Reformulated Chrastil Model	35.056^b^		− 24.638^g^ − 6.54^h^
Bartle et al., model		59.694^c^ (approximate value)	
PR EoS + vdW2 model As temperature independent		28.516^d^ (average value)	

^e^Obtained as a result of difference between the ΔH_sub_^c^ and ΔH_total_^a^.

^f^Obtained as a result between the ΔH_sub_^d^ and ΔH_total_^a^.

^g^Obtained as a result of difference between the ΔH_sub_^c^ and ΔH_total_^b^.

^h^Obtained as a result between the ΔH_sub_^d^ and ΔH_total_^b^.

The models used in correlation exercise, have a varying number of parameters and the best model is obtained with the help of Akaike’s information criterion (AIC) ^45–49^. The data used in this exercise is small (N < 40), hence corrected AIC (AIC_c_) is used for the identifying the best model. The model that gives lowest AIC_c_ value, is the best model. Table 8 indicates computed AIC and AICc values. The least AIC_c_ value − 678.88 is seen for Reddy–Garlapati model; therefore, it is considered as the best model, however, the new cluster model has also comparable performance with the best model, the corresponding AIC_c_ value is − 678.59. The highest AIC_c_ value is seen for Alwi-Garlapati, model hence it is treated as poor model for *clemastine fumarate*.

**Table 8:** Computed AIC and AIC_c_ values.

Model	SSE.10^11^	N_p_	N	AIC	AIC_c_
**Existing density models**					
Alwi–Garlapati model	2.41888	3	24	− 636.5	− 635.30
Bartel et al., model	3.29465	3	24	− 649.54	− 648.34
Bian et al., model	94.47	5	24	− 675.52	− 672.19
Chrastil model	2.69392	3	24	− 654.37	− 653.17
Reformulated Chrastil model	2.70787	3	24	− 654.25	− 653.05
Garlapati–Madras model	2.36986	5	24	− 653.45	− 650.11
Mendez–Teja model	3.2662	3	24	− 649.75	− 648.55
Sodefian et al., model	1.03868	6	24	− 671.24	− 666.30
Redddy–Garlapati model	61.5158	6	24	− 683.82	− 678.88
Mahesh–Garlapati model	2.64529	3	24	− 654.81	− 653.61
**Cluster models**					
New cluster model	82.7593	4	24	− 680.70	− 678.59
K.-W. Chen et al., model	1.10632	3	24	− 675.73	− 674.53
**EoS model**					
PR EoS model + vdW2 Mixing Rule	84.4568	5	24	− 678.21	− 674.88

## Conclusion

Solubilities of *clemastine fumarate* in ScCO_2_ at temperatures (T = 308–338 K) and pressures (P = 12–27 MPa) were reported for the first time. The measured solubilities were successfully correlated with several models; however, Tippana–Garlapati model is observed to be the best model in correlating the solubility data. The correlating ability in ascending order of various models in terms of lowest AIC_c_ values are as follows: Reddy–Garlapati model, new cluster model, PR EoS as temperature independent, K.-W. Chen et al., model, Bian et al., model, Sodefian et al., model, Mahesh–Garlapati model, Chrastil model, Reformulated Chrastil model, Garlapati–Madras model, Mendez–Teja model, Bartel et al., model, Alwi-Garlapati model. The new cluster model proposed in this work may be useful for correlating solids solubility in any SCF.

## List of symbols

*A*_1_–*A*_3_: Alwi–Garlapati parameters
*B*_1_–*B*_3_: Bartel model parameters
*D*_1_–*D*_5_: Bian et al., model parameters
*E*_1_–*E*_2_: Chrastil model parameters
*F*_1_–*F*_2_: Reformulated Chrastil model parameters
*G*_1_–*G*_5_: Garlapati–Madras model parameters
*H*_1_–*H*_3_: Mendez–Teja model parameters
*I*_1_–*I*_6_: Sodefian et al., model parameters
*J*_1_–*J*_6_: Tippana–Garlapati model parameters
*K*_1_–*K*_3_: Mahesh–Garlapati model parameters
AARD: Average absolute relative deviation
AIC: Akaike information criterion
AIC_c_: Corrected Akaike information criterion
a_ij_: EoS energy parameter
b_ij_: EoS volume correction
C: Solubility in Chrastil model
EoS: Equation of State
H_sol_: Salvation enthalpy
H_sub_: Sublimation enthalpy
H_Total_: Total enthalpy
Mscf: Molecular weight of supercritical fluid
N: Number of data points
N_p_: Number of parameters of a model
P: Total pressure
P_sub_: Sublimation pressure
PR: Peng–Robinson
P_r_: Reduced pressure
P_c_: Critical pressure
R: Universal gas constant
R^2^: Square of correlation coefficient
SSE: Sum of squares error
T: Temperature
T_c_: Critical temperature
T_r_: Reduced temperature
y_i_: Solubility in mole fraction

## Greek symbols

Δ: Difference
$\hat{\phi }_{i}^{S}$: Fugacity coefficient of the pure substance at saturation
$\hat{\phi }_{i}^{{ScCO_{2} }}$: Solute fugacity in supercritical carbon dioxide (scCO_2_)
$$\varpi$$: Acentric factor
*ρ*: Density
*ρ*_*r*_: Reduced density *ρ*_*r*_
*κ*_*ji*_: Correlation parameter
*l*_*ji*_: Correlation parameter
$\kappa ,\kappa^{\prime } ,\kappa^{\prime \prime } ,\kappa^{\prime \prime \prime }$: Association numbers in respective eqs.

## Sub and superscripts

exp: Experimental
cal: Calculated
j: Solvent/ScCO_2_
i: Solute/drug
c: Critical
r: Reduced
